# Supplementary material for: Mouse model of the human serotonin transporter-linked polymorphic region
Source: Mamm Genome. 2019 Nov 22;30(11):319–28. doi: 10.1007/s00335-019-09815-2 (PMC6884432; doi:10.1007/s00335-019-09815-2)
Supplement: Supplementary file 1 — Supplementary material 1 (PDF 318 kb) [file 335_2019_9815_MOESM1_ESM.pdf]

Mouse model of the human serotonin transporter-linked polymorphic region

Lukasz Piszczek*[1,2], Simone Memoli*[1], Angelo Raggioli[1], José Viosca[1,3], Jeanette Rientjes[4], Philip Hublitz[1,5], Weronika Czaban[1], Anna Wyrzykowska[1], and Cornelius Gross[1†]

* Equal contribution

1. Epigenetics and Neurobiology Unit, European Molecular Biology Laboratory, EMBL Rome, Monterotondo, Italy

2. Present address: Research Institute of Molecular Pathology, Vienna, Austria

3. Present address: Promotion of Health and Biomedical Research in the Valencian Region (FISABIO), Valencia, Spain.

4. Monash Genome Modification Platform (MGMP), Monash University, Clayton, Australia

5. Present address: MRC Weatherall Institute of Molecular Medicine, University of Oxford, UK

† To whom correspondence should be addressed (gross@embl.it).

Supplementary Figures and Tables

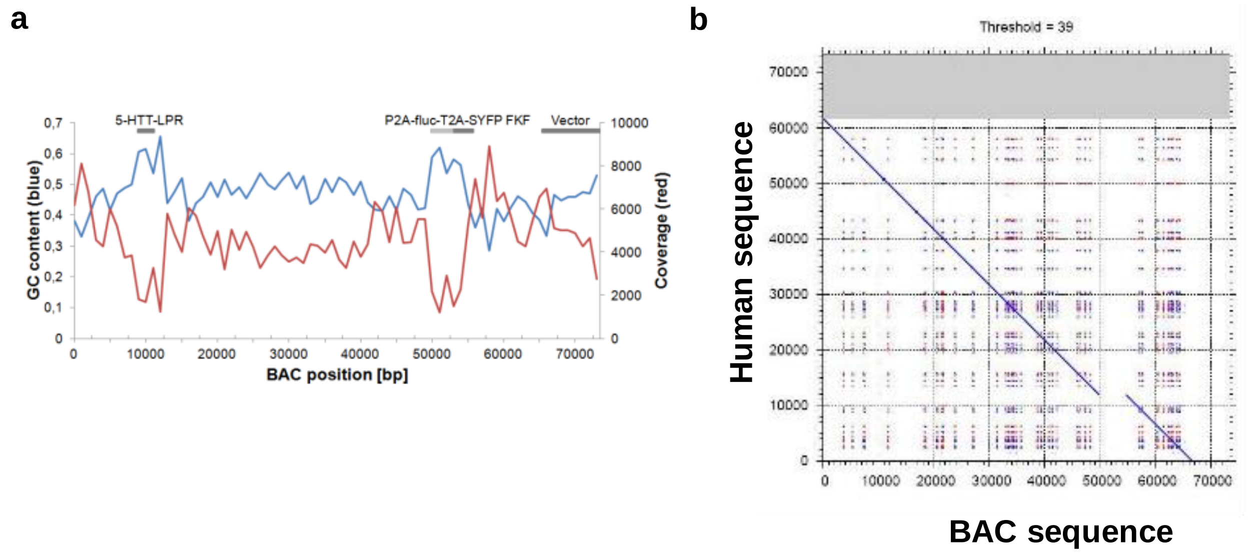

Supplementary Fig 1 Sequence of the modified 16A BAC**.** (**a**) GC content (blue) and coverage (red) of the human 5-HTT-LPR-16A targeting construct modified to co-express firefly luciferase (fLuc) and SYFP. (**b**) Comparison of the sequence with the human reference sequence showing high sequence identity (diagonal line). The gap in the diagonal line results from the co-expression cassettes at the 3’ end of the 5-HTT open reading frame in the targeting construct.

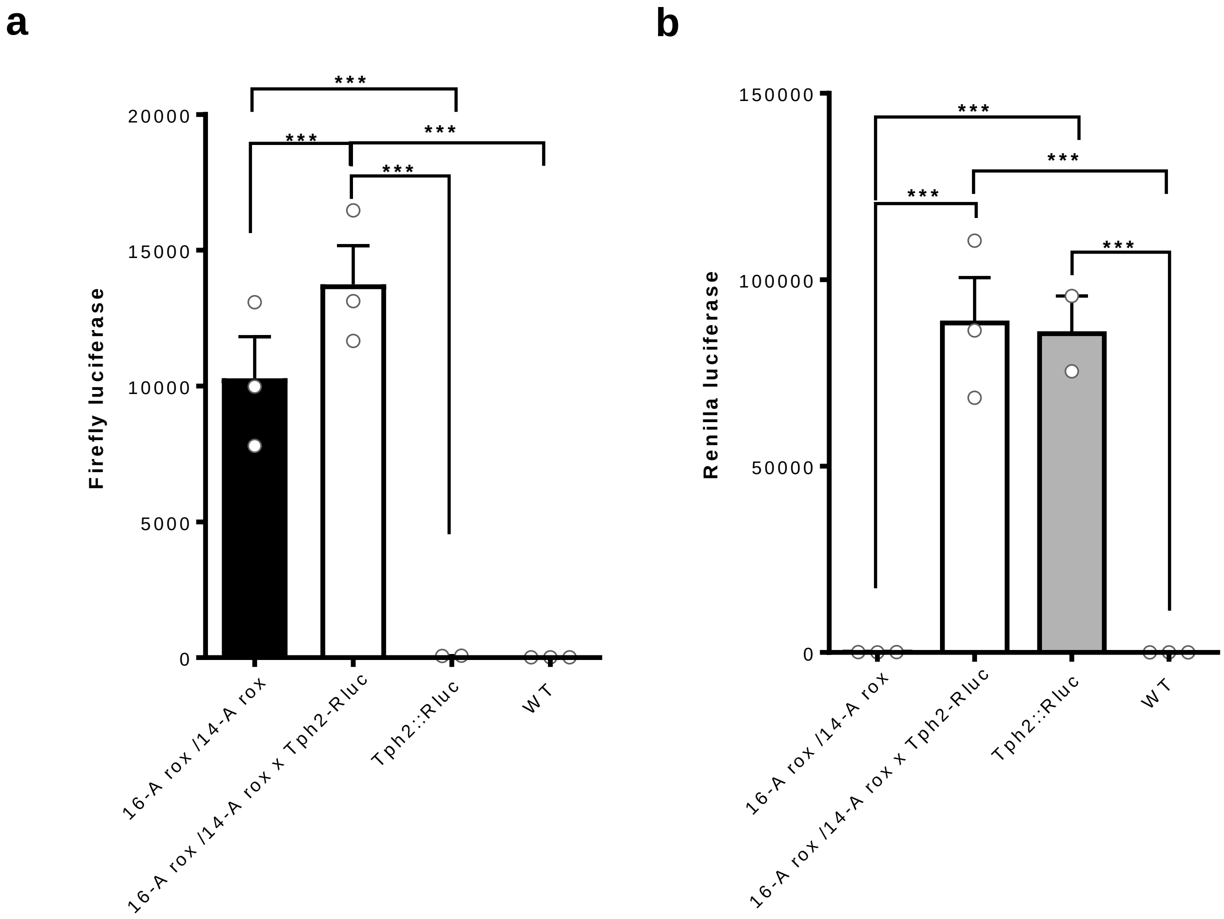

Supplementary Fig 2 Independent detection of Firefly and Renilla luciferase signal in the same sample. Absence of cross-contamination between Firefly and Renilla in the dual luciferase assay system. (**a**) Firefly luciferase signal was observed in all 16A-Rox and 14A-Rox containing mouse lines, but not in those carrying only *Tph2*::rLuc-SCFP. (**b**) Renilla luciferase signal was observed in all *Tph2*::rLuc-SCFP containing mouse lines, but not in those carrying only 16A-Rox or 14A-Rox (***p < 0.005, one-way ANOVA, Bonferroni post-hoc, compared to WT group; data shown as mean ± SEM).

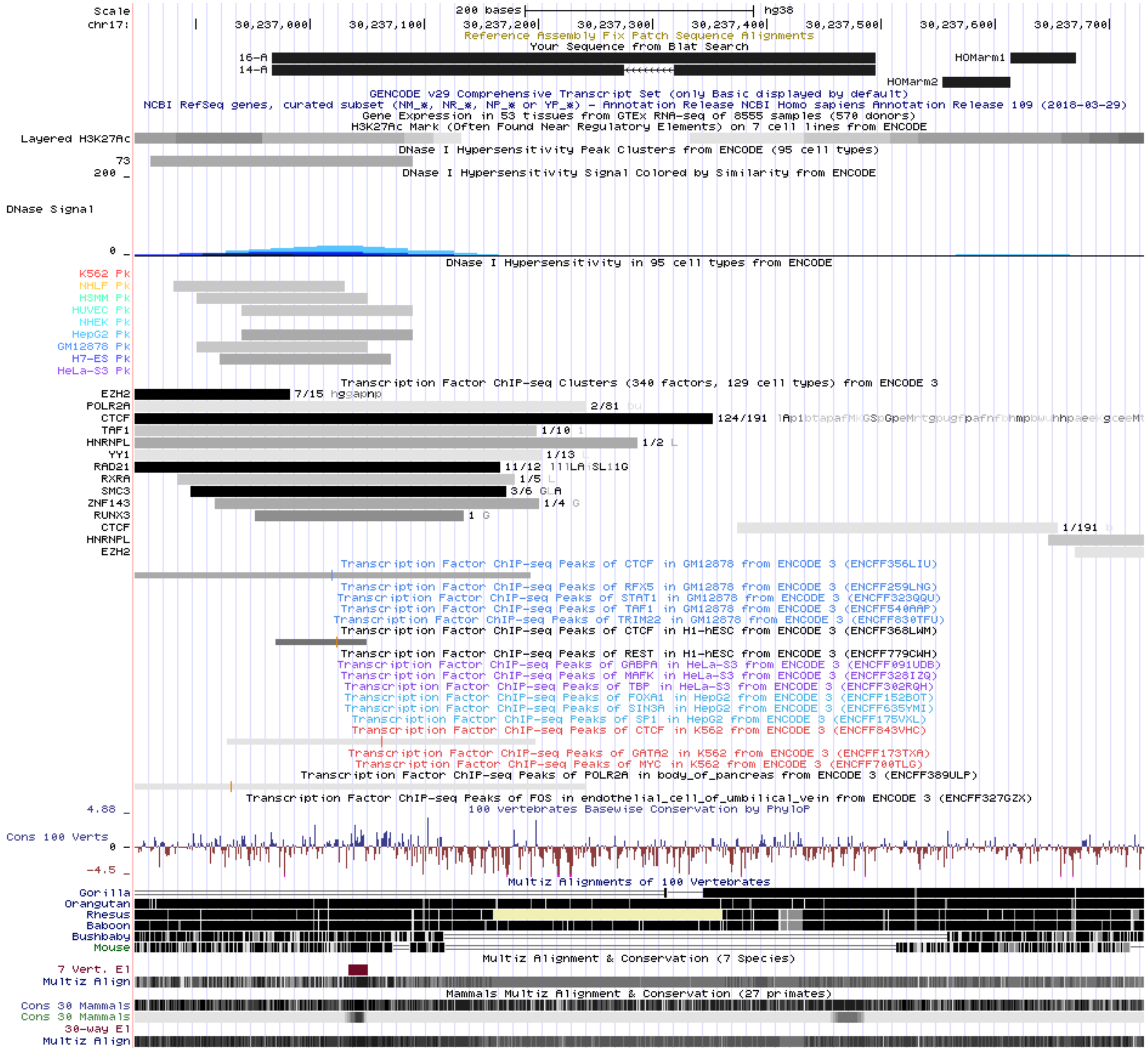

Supplementary Fig 3 Conservation of human 5-HTT-LPR. Conservation of DNA sequence surrounding the 5-HTT-LPR (16-A and 14-A; marked as black boxes). The transcription direction of 5-HTT goes towards the left side of the picture. The homology arms (HOMarm1 and HOMarm2) used to insert the Rox cassette between them are shown as black boxes on the right site. The overall conservation of the sequence in primates is relatively high. However, just between the arms there is a slight drop of sequence conservation in rhesus monkeys, species that share a similar polymorphism in the promoter region to humans. Finally, there does not seem to be any major detected binding sites from ChIP-seq transcription factor analysis nor DNase I hypersensitivity sites. Adapted from: UCSC Genome Browser (http://genome.ucsc.edu).

Supplementary Table 1 Human specific PCR primer sets used to confirm transgene integrity.

Pair no.	**Size of product (kb)**	**Primer name**	**Oligo Sequence 5'-> 3'**
1	2.4	WG hBAC 1F	AAAAGGGCTTCCCTCTTCAG
		WG hBAC 1R	GTTCTAGCAGCCAAGGATGG
2	2.5	WG hBAC 2F	TCCACAAGCTCGGGAAGACCAGG
		WG hBAC 2R	CCCCTGGGAGCCAACGGAGTA
3	2.4	WG hBAC 3F	TGCACACATTGACAGGAACA
		WG hBAC 3R	GGCAGTTTTCTATGGGCTGA
4a	1.0	WG hBAC 4Fa	TGCGTCTGGTGTAACATGGAGCTGA
		WG hBAC 4Fb	GAAGGGAATCCCCTAGGGAGGAAGT
4b	1.5	WG hBAC 4Ra	GTGGCGCCAAGGTCGTCTGGC
		WG hBAC 4Rb	TGGGGGAGGAACAGATCAGGTTCAG
5	2.5	WG hBAC 5F	TGTGGTGAGGCTGCTGCCGTA
		WG hBAC 5R	AGGAGGGGTACAGGGAGTTGCT
6	1.9	WG hBAC 6F	AGCCGTCCTCCGCTTTGGC
		WG hBAC 6R	TGCGTCACTTTGAGGCGAATAAACT
L	0.8	WG hBAC linkerR	AACGTGGGTTCGAGGCGGAGA
		WG hBAC linkerF	GCACTGTGCTCCTTTTTGACGCA
8	1.6	WG hBAC 8F	GCAGGTGGGTCCGCTTTTCC
		WG hBAC 8R	GCGGACATCCAGGGTGTTTGGG
9	2.3	WG hBAC 9F	CCCCTCAACACTCTGGTGATCCA
		WG hBAC 9R	GTGGGTGAGCTAGAGCCAGCCA
10	2.0	WG hBAC 10F	TCTGGGCGAGCACTTCTCAGCA
		WG hBAC 10R	CGAGGCTGGGAAAGAGACTTGAGA
11	2.5	WG hBAC 11F	AGCTGTGTGTGGACATGTTCCCATG
		WG hBAC 11R	CCTGCCTGGGAGAGGATAGCCC
12	2.5	WG hBAC 12F	GTGGGCCTCAGTTTCCCTGCTAG
		WG hBAC 12R	ATAGCCAGGAGCAACCCGTACCTG
13	2.5	WG hBAC 13F	TTACTGCTTCGGGCGGCACCA
		WG hBAC 13R	TTTATGCTACTGCCTGGCCTTGGC
14	1.8	WG hBAC 14F	CCTTCCGTAGACCCTCTGGGCC
		WG hBAC 14R	AACGGCACTGCTGCTCACCAT
15	1.4	WG hBAC 15F	TCCAGTGTCATCTCAGCTAGGCAG
		WG hBAC 15R	CTCCCGCACCAGGACTTGGA
16	1.4	WG hBAC 16F	TCCAGCCTGTGCAAACCTTGGTGAT
		WG hBAC 16R	GCTTCACAACCCGGAGAGCCTC
17	2.3	WG hBAC 17F	ACACGGCACTCTATCCCAGCG
		WG hBAC 17R	CAGCTGCAACTCTCTGTGAGTCACC
18	2.4	WG hBAC 18F	TTGGAGGAAGGCCATCACGAGAACA
		WG hBAC 18R	AAGCACATTTGGCCAACACCCTGG
19	2.3	WG hBAC 19F	TACCTCAAGTGCTCCACGGCCT
		WG hBAC 19R	GGACCCCAAAGCCCGGACCAA

Pair no.	Size of product (kb)	Primer name	Oligo Sequence 5'-> 3'
20	1.8	WG hBAC 20F	AGGAGTGGCGACCCTGTTGGT
		WG hBAC 20R	CACTGCAGCCTTAACCTCCCGG
21	1.1	WG hBAC 21F	CAGGAGGATTGCTTGAGCCCGG
		WG hBAC 21R	GCGTCTTTGGCCACCTCAGAC
22	2.1	WG hBAC 22F	GCGGCCCCTTGGGTTTTCCC
		WG hBAC 22R	CAGTTCCCCGGCTCGCTGG
23	0.8	WG hBAC 23F	AATTATGGCGCCTACAGGCCGG
		WG hBAC 23R	GCGGTAAAATGCTGACAGCCCCTG
24	0.8	WG hbAC 24F	GGCTTGGAGGGGGTGATCACG
		WG hBAC 24R	TTGAACACCTGCTGTGGACACGA
25	1.7	WG hBAC 25F	GGCTGTCAGCATTTTACCGCAGA
		WG hBAC 25R	AGCCCTGTGTGCAACCCAAAATGT
26	2.4	WG hBAC 26F	ACCTCCCGATGATGGGTCTGTAAAA
		WG hBAC 26R	AGGGCCCCCATGAAGAGCATAGC
27	1.6	WG hBAC 27F	AGTGGTTATAGTTCTGGCACTCCCA
		WG hBAC 27R	TCTGGTAGGATGGGCTAGGCGA
28	2.3	WG hBAC 28F	TCTTGTCTCTGTGACACTGCATGTG
		WG hBAC 28R	TTACCAACTTCTGTACCCAAGCTGC
29	1.8	WG hBAC 29F	ATGATGAGGACCTGTGGCAGGC
		WG hBAC 29R	AATTAGCCCACCTCCGTTCTCAGGC
30	2.0	WG hBAC 30F	GTACCTTGGGAGGTAAATGGGCAGG
		WG hBAC 30R	CGCAAGACTTCCAGACCGTGGGT
31	2.1	WG hBAC 31F	ACGCCGAGGTGGATGGGTCA
		WG hBAC 31R	TGCGGCCACAAAGGGCTTGG
32	2.4	WG hBAC 32F	TTTCAAAGCCTCTGCAGTGTGGCT
		WG hBAC 32R	TTGCCGTGTTCCAGCACGACG
33	2.4	WG hBAC 33F	GCGCCCCACTGTCTAAGGAGGT
		WG hBAC 33R	TTACGGTATCGCCGCTCCCGA
34	2.1	WG hBAC 34F	AGAAACGCGGGCGTATTGGCC
		WG hBAC 34R	ATCTTCCTTTCCTGATGCCACAGCA
35	1.8	WG hBAC 35F	CCAGTTCTGATGAGGCACGCC
		WG hBAC 35R	GGGTGGGGAGAATCCATATCCGA
36	1.3	WG hBAC 36F	TCCAGACAGCAGAGGAAACATCCT
		WG hBAC 36R	ATTAGCTTGGTCCCCAGGTTTGTCT
37	1.0	WG hBAC 37F	AGTGAGCCAAACGTTCCACTGCA
		WG hBAC 37R	TGATGCCATCTATTCTCTCCCCCAA
38	1.6	WG hBAC 38F	TGCCAAGCTGCTCTACCACCAATG
		WG hBAC 38R	GGCAGGAGGTCTGTTAGGAACTGGA
39	1.9	WG hBAC 39F	CTTTTTGGCACCGGGACCGGT
		WG hBAC 39R	GGTCGAGGCTGTAGTGAGCTGTGA
40	1.1	WG hBAC 40F	ACTGCTGCATGACTGTCAACTCATC
		WG hBAC 40R	ATCCAGTGAGAGGGCTGAGGATCA

Pair no.	**Size of product (kb)**	**Primer name**	**Oligo Sequence 5'-> 3'**
41	1.4	WG hBAC 41F	TTGCCCAGGCGGTTCTCCTG
		WG hBAC 41R	TGTCGCCCAGGCTAGAGTGC
42	1.2	WG hBAC 42F	ATTCCTGGTGCATCTGTGGAACACG
		WG hBAC 42R	GGAACTTCTCCCTCCCCTCTATGGC
43	1.3	WG hBAC 43F	AATACCGTTGCTTCGCATCCTCATC
		WG hBAC 43R	TTCCAGTTGCCCCACATCTTGCT
44	1.2	WG hBAC 44F	AGGCAGGCGAAATTGCTTGAGCC
		WG hBAC 44R	TCCACGGGTTCAAATGGGGCAG

Supplementary Data 1. 5-HTT-LPR modified sequence

Key part of the modified 5-HTT-LPR: Homology arms, roxP sequence, 5-HTT-LPR (16-A, and sequence not present in 14-A variant), Exon 1A of the 5-HTT.

CAGGGCAGGAGGAAGAGAAACACCTGTTTAGAGAGAGGGGGAGGGTCTGGGCCAGTTGTGTGCGGAC
TCTGCCATGGAGGCAGGGGGAACCAGGGCGGGGCCGTCTGTGTAATTCTAATGTCATTTGCTAATGGT
GGTTGCCCGCAGCTGGGGAGCAAGAGGGGAGGAGATGGGACCACCCGGCCAGAGAGAGGAGGGGTC
GAAGGCCCCCTTGGAGGAAGGGCCCAGCGCACAGGGCCTGCAAGGAGTTGGCCCCTTCCCCACCCACA
GCTGGGCAGTGCCCACTGAGATCATCCTCCCTACTTCACAAACAGAGACACAAAGGCCAGACAGCTTG
AGGTTAAGGAGCTCGGTCAAGCCCACACCACAGTACAGGGTGGGGGCCGGGATTTGACCCAGGTCCAC
AGGGTGCCCACTCTCTAGGCAGCACCTGCACAGAACAGGGCAGTGCCATAGGGAGGGGCTGCGCGCTG
GGCAGGAATGCATCTTCGGCTAGAAAGAGTCTAGAAGGAAGATCAGAAGTGGCGAGACTGCACAATT
AAGAAGTGCTAAGAAGTCACACTGGACAGACCTGGTCTTGGTAGCCAAGCGCTGAAGTCACCCCTGCA
GCCGTCCTCCGCTTTGGCGCCTCTTCCCAGCGTCCCTGCCCCCCTCCTTTGGCCCTCCTGGAAAGGACAC
TTTGCGTTTTCTGTTGCCCTTGCCTATACAGCACAAACATGCTCATTTAAG**TAACTTTAAATAATGCCA**
ATTATTTAAAGTTAAAGTGGAACGTGGGAGGCAGCAGACAACTGTGTTCATCTGAAAGGAGGAGGCC
CCACTCCCGTGCAGGCGCTGCCCCTGGGGGTGAAATTCCCAAGCTTGTTGGGGATTCTCCCGC**CTGGC**
GTTGCCGCTCTGAATGCCAGCACCTAACCCCTAATGTCCCTACTGCAGCCCTCCCAGCATCCCCC
CTGCAACCTCCCAGCAACTCCCTGTACCCCTCCTAGGATCGCTCCTGCATCCCCCATTATCCCCC
CCTTCACCCCTCGCGGCATCCCCCCTGCACCCCCAGCATCCCCCCTGCAGCCCCCCCAGCATCTC
CCCTGCACCCCCAGCATCCCCCCTGCAGCCCTTCCAGCATCCCCCTGCACCTCTCCCAGGATCTC
CCCTGCAACCCCCATTATCCCCCCTGCACCCCTCGCAGTATCCCCCCTGCACCCCCCAGCATCCC
CCCATGCACCCCCGGCATCCCCCCTGCACCCCTCCAGCATTCTCCTTGCACCCTACCAGTATTCC
CCCGCATCCCGGCCTCCAAGCCTCCCGCCCACCTTGCGGTCCCCGCCCTGGCGTCTAGGTGGCA
CCAGAATCCCGCGCGGACTCCACCCGCTGGGAGCTGCCCTCGCTTGCCCGTGGTTGTCCAGCTC
AGTCCCTCTAGACGCTCAGCCCAACCGGCCGCACAGTTTTCAGGGGTCAGTTCCTCCAAGTACAAGGG
GCGGTGGCTTCTCTGGAGCTGCAAACTTGTCACTGCTATTTCCTTTCGGTCTTCTACTTCCTATCGTTCCT
GGCCTCCTCTTGGGGAGAGGTAGAGCCCTCTCCTTTCCGCCTCAGGGACAACCCAAAGCAAGTACTGC
ATGTGCCCTTTTTAAAGTTTTAAATAATTTTAGCAAAAAGGATATTAACATTAAATCAATTTTTAAACTT
TTTGAAAAAATTATCAAAACTACATGCACATGGTTCAAAACAATAGGCTCCTGCTGGGCCCTTTCAGAT
AATTCAAATTGTCACCAGGTTGGAGTGCAGTGGTTCGATCACGGCTCACTGCAGCCTCGACCTCCCGGG
CTCAGCTGATCCTCCACCTCAGCCTCCTGAGTAGCTGGGAACACAAGCGCGAGCAACCACGCCCGGCT
AATTAAAAAAATTTTTTTTCTAGAGATGGGGTCTTGCTGTGTTGCCCAGGCTGGTCTTGAATTCCTGGG
CTCAAGCAATCCTCCCGCCTCAGCCTCCCAAAGCACTGTGCTCCTTTTTGACGCAGCTTTGAACTGTAG
CTGGTTAACAAAATGAGAACCAGTTCTTCATTCCTTCATTGTGGAAGTCTTTATTGTGAGACTCTGGGG
ACGGAGAGGAATTAGACAAGGGCCTCTAAGCTGAGCTCACATCCCAGCCGGTCAGTCAGATAAACGCA
TGGGTATCGAGTACTGCTAGGTCCCAGGAAGAAAGAGAGAGCAGCTTTCGGGATGGGGACGATGGGG
AGGTGTCCGAGGTCAAGAGAAAGCGGCACGAGCAGACCCCTGTGTGCCGTCCTGTGGGCGCGGGGCG
GCAGGGGAGGCGCACACCTGCTCCTTTGTGCAGCCTCCCCCCTCCCGCAAAGTTAAAGAGCAGGAAAG
TCAGGATTCCTCGCTCGGCCCTGCCCTGCCGGCTGCTCCGCGCTCCGCTCCTCCCTGCGAGCGTGTGTGT
GTGTCGGGGGTCCCTCCCCTCCTGGCTCTGGGGTCGGGCGCGCACCCCGCCCCGTAGCGCGGCCCCTCC
CTGGCGAGCGCAACCCCATCCAGCGGGAGCGCGGAGCCGCGGCCGCGGGGAAGCATTAAGTTTATTCG
CCTCAAAGTGACGCAAAAATTCTTCAAGAGCTCTTTGGCGGCGGCTATCTAGAGATCAGACCATGTGA
GGGCCCGCGGGTACAAATACGGCCGCGCCGGCGCCCCTCCGC**ACAGCCAGCGCCGCCGGGTGCCTC**
GAGGGCGCGAGGCCAGCCCGCCTGCCCAGCCCGGGACCAGCCTCCCCGCGCAGCCTGGCAG

Supplementary Data 2. 3’ end of the modified 5-HTT gene

Key part of the modified 5-HTT gene: 3’end of 5-HTT, P2A sequence, firefly luciferase, T2A sequence, SYFP2, FRT-NEO-FRT cassette, FRT sites

AACTCGCTCTTAGATGTTATTAAAGTGTTATTTAAGCTTTGTTTTAATATTAATGTTGACTATTTTTGCA
AGTTTTAAAAATTACAAGGATGTTTATAACATTGTATTTTCTTCCCAATAGCGTATTATTAAAAGTATTA
CCCCAGAAACACCAACAGAAATTCCTTGTGGGACATCCGCTTGAATGCTGTG**GGCTCCGGAGCCAC**
GAACTTCTCTCTGTTAAAGCAAGCAGGAGACGTGGAAGAAAACCCCGGTCCCATGGCCGATGCT
AAGAACATTAAGAAGGGCCCTGCTCCCTTCTACCCTCTGGAGGATGGCACCGCTGGCGAGCAGC
TGCACAAGGCCATGAAGAGGTATGCCCTGGTGCCTGGCACCATTGCCTTCACCGATGCCCACAT
TGAGGTGGACATCACCTATGCCGAGTACTTCGAGATGTCTGTGCGCCTGGCCGAGGCCATGAAG
AGGTACGGCCTGAACACCAACCACCGCATCGTGGTGTGCTCTGAGAACTCTCTGCAGTTCTTCAT
GCCAGTGCTGGGCGCCCTGTTCATCGGAGTGGCCGTGGCCCCTGCTAACGACATTTACAACGAG
CGCGAGCTGCTGAACAGCATGGGCATTTCTCAGCCTACCGTGGTGTTCGTGTCTAAGAAGGGCC
TGCAGAAGATCCTGAACGTGCAGAAGAAGCTGCCTATCATCCAGAAGATCATCATCATGGACTCT
AAGACCGACTACCAGGGCTTCCAGAGCATGTACACATTCGTGACATCTCATCTGCCTCCTGGCTT
CAACGAGTACGACTTCGTGCCAGAGTCTTTCGACAGGGACAAAACCATTGCCCTGATCATGAACA
GCTCTGGGTCTACCGGCCTGCCTAAGGGCGTGGCCCTGCCTCATCGCACCGCCTGTGTGCGCTT
CTCTCACGCCCGCGACCCTATTTTCGGCAACCAGATCATCCCCGACACCGCTATTCTGAGCGTGG
TGCCATTCCACCACGGCTTCGGCATGTTCACCACCCTGGGCTACCTGATTTGCGGCTTTCGGGTG
GTGCTGATGTACCGCTTCGAGGAGGAGCTGTTCCTGCGCAGCCTGCAAGACTACAAAATTCAGT
CTGCCCTGCTGGTGCCAACCCTGTTCAGCTTCTTCGCTAAGAGCACCCTGATCGACAAGTACGAC
CTGTCTAACCTGCACGAGATTGCCTCTGGCGGCGCCCCACTGTCTAAGGAGGTGGGCGAAGCCG
TGGCCAAGCGCTTTCATCTGCCAGGCATCCGCCAGGGCTACGGCCTGACCGAGACAACCAGCGC
CATTCTGATTACCCCAGAGGGCGACGACAAGCCTGGCGCCGTGGGCAAGGTGGTGCCATTCTTC
GAGGCCAAGGTGGTGGACCTGGACACCGGCAAGACCCTGGGAGTGAACCAGCGCGGCGAGCTG
TGTGTGCGCGGCCCTATGATTATGTCCGGCTACGTGAATAACCCTGAGGCCACAAACGCCCTGA
TCGACAAGGACGGCTGGCTGCACTCTGGCGACATTGCCTACTGGGACGAGGACGAGCACTTCTT
CATCGTGGACCGCCTGAAGTCTCTGATCAAGTACAAGGGCTACCAGGTGGCCCCAGCCGAGCTG
GAGTCTATCCTGCTGCAGCACCCTAACATTTTCGACGCCGGAGTGGCCGGCCTGCCCGACGACG
ATGCCGGCGAGCTGCCTGCCGCCGTCGTCGTGCTGGAACACGGCAAGACCATGACCGAGAAGGA
GATCGTGGACTATGTGGCCAGCCAGGTGACAACCGCCAAGAAGCTGCGCGGCGGAGTGGTGTTC
GTGGACGAGGTGCCCAAGGGCCTGACCGGCAAGCTGGACGCCCGCAAGATCCGCGAGATCCTG
ATCAAGGCTAAGAAAGGCGGCAAGATCGCCGTGGGATCCGGA**GAGGGCAGAGGAAGTCTTCTAA**
CATGCGGTGACGTGGAGGAGAATCCCGGCCCTATGGTGAGCAAGGGCGAGGAGCTGTTCACCG
GGGTGGTGCCCATCCTGGTCGAGCTGGACGGCGACGTAAACGGCCACAAGTTCAGCGTGTCCGG
CGAGGGCGAGGGCGATGCCACCTACGGCAAGCTGACCCTGAAGCTGATCTGCACCACCGGCAAG
CTGCCCGTGCCCTGGCCCACCCTCGTGACCACCCTGGGCTACGGCGTGCAGTGCTTCGCCCGCT
ACCCCGACCACATGAAGCAGCACGACTTCTTCAAGTCCGCCATGCCCGAAGGCTACGTCCAGGA
GCGCACCATCTTCTTCAAGGACGACGGCAACTACAAGACCCGCGCCGAGGTGAAGTTCGAGGGC
GACACCCTGGTGAACCGCATCGAGCTGAAGGGCATCGACTTCAAGGAGGACGGCAACATCCTGG
GGCACAAGCTGGAGTACAACTACAACAGCCACAACGTCTATATCACCGCCGACAAGCAGAAGAA
CGGCATCAAGGCCAACTTCAAGATCCGCCACAACATCGAGGACGGCGGCGTGCAGCTCGCCGAC
CACTACCAGCAGAACACCCCCATCGGCGACGGCCCCGTGCTGCTGCCCGACAACCACTACCTGA
GCTACCAGTCCAAGCTGAGCAAAGACCCCAACGAGAAGCGCGATCACATGGTCCTGCTGGAGTT
CGTGACCGCCGCCGGGATCACTCTCGGCATGGACGAGCTGTACAAGTAATTCTAGCCGCTTCGAG
CAGACATGATAAGATACATTGATGAGTTTGGACAAACCACAACTAGAATGCAGTGAAAAAAATGCTTT
ATTTGTGAAATTTGTGATGCTATTGCTTTATTTGTAACCATTATAAGCTGCAATAAACAAGTTAACAAC
AACAATTGCATTCATTTTATGTTTCAGGTTCAGGGGGAGGTGTGGGAGGTTTTTTAAAGCAAGTAAAAC
CTCTACAAATGTGGTAAAATCGATAAGGATCCAGGAAGGGCGAATTCCAGCACACTGGCGGCCGTTAC
TAGTGGATCCGAGCTCGGTACCCTCACTATAGGGCGAATTGGAGCTCCACCGCGGTGGCGGCCGCTTA
GTTTAAACTCGAG**GAAGTTCCTATACTTTCTAGAGAATAGGAACTTC**GGATCCTGGCAGGGCCTGCC
GCCCCGACGTTGGCTGCGAGCCCTGGGCCTTCACCCGAACTTGGGGGGTGGGGTGGGGAAAAGGAAG
AAACGCGGGCGTATTGGCCCCAATGGGGTCTCGGTGGGGTATCGACAGAGTGCCAGCCCTGGGACCGA
ACCCCGCGTTTATGAACAAACGACCCAACACCGTGCGTTTTATTCTGTCTTTTTATTGCCGTCATAGCGC
GGGTTCCTTCCGGTATTGTCTCCTTCCGTGTTTCAGTTAGCCTCCCCCTAGGGTGGGCGAAGAACTCCA

GCATGAGATCCCCGCGCTGGAGGATCATCCAGCCGGCGTCCCGGAAAACGATTCCGAAGCCCAACCTT
TCATAGAAGGCGGCGGTGGAATCGAAATCTCGTGATGGCAGGTTGGGCGTCGCTTGGTCGGTCATTTC
GAACCCCAGAGTCCCGC**TCAGAAGAACTCGTCAAGAAGGCGATAGAAGGCGATGCGCTGCGAATC**
GGGAGCGGCGATACCGTAAAGCACGAGGAAGCGGTCAGCCCATTCGCCGCCAAGCTCTTCAGCA
ATATCACGGGTAGCCAACGCTATGTCCTGATAGCGGTCCGCCACACCCAGCCGGCCACAGTCGA
TGAATCCAGAAAAGCGGCCATTTTCCACCATGATATTCGGCAAGCAGGCATCGCCATGGGTCAC
GACGAGATCCTCGCCGTCGGGCATGCTCGCCTTGAGCCTGGCGAACAGTTCGGCTGGCGCGAGC
CCCTGATGCTCTTCGTCCAGATCATCCTGATCGACAAGACCGGCTTCCATCCGAGTACGTGCTCG
CTCGATGCGATGTTTCGCTTGGTGGTCGAATGGGCAGGTAGCCGGATCAAGCGTATGCAGCCGC
CGCATTGCATCAGCCATGATGGATACTTTCTCGGCAGGAGCAAGGTGAGATGACAGGAGATCCT
GCCCCGGCACTTCGCCCAATAGCAGCCAGTCCCTTCCCGCTTCAGTGACAACGTCGAGCACAGC
TGCGCAAGGAACGCCCGTCGTGGCCAGCCACGATAGCCGCGCTGCCTCGTCCTGCAGTTCATTC
AGGGCACCGGACAGGTCGGTCTTGACAAAAAGAACCGGGCGCCCCTGCGCTGACAGCCGGAAC
ACGGCGGCATCAGAGCAGCCGATTGTCTGTTGTGCCCAGTCATAGCCGAATAGCCTCTCCACCC
AAGCGGCCGGAGAACCTGCGTGCAATCCATCTTGTTCAATGGCCGATCCCATGGTTTAGTTCCTC
ACCTTGTCGTATTATACTATGCCGATATACTATGCCGATGATTAATTGTCAACACGTGCTGCTGC
AGGTCGAAAGGCCCGGAGATGAGGAAGAGGAGAACAGCGCGGCAGACGTGCGCTTTTGAAGCG
TGCAGAATGCCGGGCCTCCGGAGGACCTTCGGGCGCCCGCCCCGCCCCTGAGCCCGCCCCTGAG
CCCGCCCCCGGACCCACCCCTTCCCAGCCTCTGAGCCCAGAAAGCGAAGGAGCAAAGCTGCTAT
TGGCCGCTGCCCCAAAGGCCTACCCGCTTCCATTGCTCAGCGGTGCTGTCCATCTGCACGAGAC
TAGTGAGACGTGCTACTTCCATTTGTCACGTCCTGCACGACGCGAGCTGCGGGGCGGGGGGGAA
CTTCCTGACTAGGGGAGGAGTAGAAGGTGGCGCGAAGGGGCCACCAAAGAACGGAGCCGGTTGGCG
CCTACCGGTGGATGTGGAATGTGTGCGAGGCCAGAGGCCACTTGTGTAGCGCCAAGTGCCCAGCGGGG
CTGCTAAAGCGCATGCTCCAGACTGCCTTGGGAAAAGCGCCTCCCCTACCCGGTAGCCCGAAAAGTGC
CACCTAAGCTT**GAAGTTCCTATACTTTCTAGAGAATAGGAACTT**CCAGGTACCCAGCTTTTGTTCCCT
TTAGTGAGGGTTAATTGCGCGCTTGGCGTAATCATGGTCATAGCTGTTTCCTGTG
